# Suggestions for Radiation Oncologists during the COVID-19 Pandemic

**DOI:** 10.1155/2020/4892382

**Published:** 2020-05-26

**Authors:** Pierfrancesco Franco, Lofti Kochbati, Marco Siano, Berardino De Bari

**Affiliations:** ^1^Department of Oncology, Radiation Oncology, University of Turin, Turin, Italy; ^2^Department of Radiation Oncology, Ariana, Tunis El Manar University, Tunisia; ^3^Interdisciplinary Cancer Service-SIC, Riviera-Chablais Hospital, Rennaz, Switzerland; ^4^Department of Radiation Oncology, Réseal Hospitalier Neuchâtelois, La-Chaux-de Fonds, Switzerland

## Abstract

SARS-CoV-2 pandemic and COVID-19 diffusion have recently become an international public health emergency. Cancer patients, as a frail population, are particularly exposed to the risk related to infections. The clinical decision-making process and the organizational workflow of radiotherapy department should be revised in the light of the critical situation. We herein provide practical suggestions derived from the available literature and discussed during an online session held within the e-learning educational program of the European School of Oncology on March 31st 2020.

## 1. Introduction

At the end of December 2019, in the city of Wuhan Hubei Province, China, the observation of a cluster of patients with severe respiratory syndrome lead to discovery of the SARS-CoV-2 infection, which is presently known to be responsible for COVID-19 [1]. By mid-February, more than 60.000 cases were reported in China, and by mid-March, around 170.000 cases were observed worldwide, prompting the World Health Organization (WHO) to label the coronavirus outbreak as a pandemic on March 11th, 2020 [2]. COVID-19 hence became an international public health emergency. Starting from the early phases of the outbreak, it has been noted that COVID-19 can be extremely severe in high-risk patients, including the elderly population and those with comorbidities. The case fatality rate for Chinese patients aged between 70 and 79 years was reported to be 8.0% and 14.8% for those aged ≥80 [3]. In Italy, one of the most affected countries outside China, the case fatality rates for the same age categories were reported as high as 12.8% and 20.2% [4]. Cancer patients, being a frail population, are particularly exposed to the risk of COVID-19. Data from Wuhan highlighted a case fatality rate for cancer patients up to 5.6% compared to 2.1% of the general population. Moreover, they bear a 5-fold relative risk of severe respiratory manifestations, requiring invasive ventilation and finally leading to death [5, 6].

Radiation therapy is a mainstay pillar for cancer treatment as around 50% of cancer patients would require radiation during the course of their disease [7]. During standard periods, radiotherapy would be considered as a “life-saving” procedure, and consequently, efforts should be placed to ensure its access to all cancer patients [8]. In such a critical situation, as the COVID-19 pandemic is, and given the fractionated nature of radiotherapy treatments, the risk-benefit ratio may be different and specific decisions and actions could be taken, particularly in case of concurrent chemotherapy administration, calling for shared and interdisciplinary decision-making [9]. Another point is the burden that the pandemic situation may have on radiotherapy departments worldwide, with the need to differently allocate resources, selectively screen the patients on a daily basis for treatments, deal with a shortage in workforce, and carefully plan for strategies to properly address treatment interruptions or delays [10]. Finally, the safety of healthcare workers and noninfected patients should be prioritized. Different contributions have been recently published, including editorials, perspectives, statements, and recommendations. We herein would like to wrap up the main findings, inspired by the online session that was held on March 31st, 2020, within the European School of Oncology online education platform e-ESO (http://www.eso.net).

## 2. Clinical Indications to Radiotherapy

During standard periods, oncologists are supposed to rely on robust data supported by high-quality evidence in the therapeutic decision-making process. In such a dramatic framework, as the COVID-19 pandemic is, different considerations may drive clinical practice, with the possibility to offer less typical treatment regimens, supported by lower quality of evidence [11]. Focused attention should be placed on the proper assessment of the risk-benefit ratio, balancing the exposure to infection risk with the potential benefit derived from radiotherapy treatment. For each patient, the clinical setting should be evaluated together with the purpose to deliver radiotherapy (definitive/neoadjuvant vs. adjuvant vs. palliative radiation) [11]. Radiotherapy omission could be evaluated for

1. low-risk patients (examples: (a) low-risk luminal A breast cancer in patients aged ≥65-70 after breast conservation if undergoing endocrine therapy; (b) low or favorable intermediate risk prostate cancer willing to undergo active surveillance
2. clinical contexts with a predicted small benefit derived from radiotherapy (examples: (a) glioblastoma in methylated patients aged >60; (b) unresectable pancreatic adenocarcinoma)
3. palliative settings amenable to systemic options (uncomplicated metastases, painful metastases manageable with tailored analgesics) [11]

Whenever radiotherapy is deemed indicated, clinicians need to consider that “less may be better” and employ hypofractionated schedules that already have shown noninferiority compared to standard fractionation in well-designed trials, such as in breast (for whole and partial breast irradiation and nodal irradiation), prostate (moderate hypofractionation or extreme hypofractionated schedules), and rectal (short course radiation vs. long-course chemoradiation) cancers and gliomas (moderate hypofractionated schedules) [12].

## 3. The Example of Breast Cancer

Breast cancer is a paradigmatic example on how the radiotherapy decision-making process can be adapted to this particular situation and a proof of the versatility of radiation oncology treatment strategies, as pointed out in the guidelines for breast cancer during the COVID-19 pandemic [13]. Radiotherapy could be safely omitted, after breast conservation, for low-risk ductal in situ carcinoma or infiltrative disease with low-risk features (size ≤ 30 mm, node negative, G1-G2, clear resection margins, estrogen receptor positive, HER2 negative, in patients aged >65 planned to receive hormonal manipulation) [13, 14]. When whole breast radiotherapy is indicated, 5 fractions can be used for node-negative patients not requiring a boost (28-30 Gy in once weekly fractions over 5 weeks as per FAST trial or 26 Gy in 5 daily fractions in 1 week as per FAST Forward trial). Boost dose to the tumor bed should be omitted unless for young patients (≤40 years) and/or for those having high-risk factors for local recurrence [15]. Nodal radiotherapy can be omitted for postmenopausal women after breast conservation in case of 1-2 macrometastases at sentinel lymph node biopsy and low-risk features (size ≤ 2 cm, G1-G1, estrogen receptor-positive and HER2-negative disease) [13].

## 4. Management of Patients with COVID-19

This is a challenging situation not only in terms of staff safety but also considering the need to preserve the care and well-being of other patients within the radiotherapy department. A hypothetical triage decision tree has been proposed by colleagues from the United States [10].

For patients having a positive test for SARS-CoV-2 referred for radiotherapy, the first steps should include

1. the assessment of the adequateness of Personal Protection Equipment (PPE) for all the personnel exposed
2. the confirmation of the appropriate isolation policy with healthcare authorities, and
3. the evaluation of the appropriateness of the referral and the cogency of treatment

If timely initiation of radiotherapy is deemed crucial for the patient, clinicians should

1. use PPE in all radiotherapy steps
2. observe all hospital authorities' indications
3. properly sanitize/sterilize or discard all the radiotherapy equipment in contact with the patient
4. use the shortest hypofractionated schedules whenever reasonable from a clinical perspective

For patients to whom radiotherapy initiation is important, but not crucial, clinicians should consider delaying treatment after the emergency period and, if possible, after the recommended social-distancing policies are lifted and use hypofractionation when treating the patient. For patients whose radiotherapy start can be reasonably delayed, treating physicians should pursue this policy [10].

For low- or middle-income countries, adequate protection measures may not be trivial to implement. If so, a practical decision may be to stop treatment for SARS-CoV-2-positive patients and restart the treatment whenever possible with adequate compensation for the protraction in overall treatment time. Specific recommendations for low- or middle-income countries are urgently needed.

## 5. Protecting the Staff and Patients

Reducing the risk of infection for both the staff and patients is crucial. Radiotherapy departments should follow the national directives and the procedures established by the local authorities for infection control, which should be proactively involved in the inspection of all the processes within the department [11, 16]. As basic principles, the staff should wash hands before and after contact with the patient. Patients should also wash their hands or use alcohol hand rub before entering and leaving the department. Temperature screening for staff, patients, and visitors is advisable in compliance with local authorities' suggestions. Limiting the access to the radiotherapy department to patients and necessary caregivers could also be suggested. A rationalization process to limit patient contact may also be helpful, creating specific pathways for infected and/or suspected cases within the department and proactively minimizing the interaction with staff and other patients [11, 17]. Restriction of staff movements around the various sections of the departments is advisable, employing, if possible, limited functional teams to accomplish the different professional tasks. Physical preparation of the department, with eventual decontamination, should be favored. For patients with high mucosal or aerosol output, wearing a mask should be suggested to decrease the risk of contamination [11].

## 6. A Help from Technology

Technology can be a very helpful tool to enhance suppressive social distancing, by helping to implement remote working [11, 18]. All the working typologies that could be exploited from home, in compliance with local governance and information technology regulation, should be fostered. As an example, most of the processes involving the personnel working in the medical physics department can be assigned to smart working, including treatment planning, dosimetry assessment, equipment quality assurance, and dosimetry check [16]. It means that all electronic charts and treatment planning data could be accessible remotely and installed on the personal laptops of the staff members. Moreover, a proactive adaptation of the available technologies should be considered by the IT staff of the hospital including, for example, an increase in the VPN traffic capability. For academic departments, all the teaching and training activities could be exploited remotely, using online platforms and live-streaming sessions [19–21].

## 7. Conclusion

The global challenges for the healthcare system of the COVID-19 pandemic have no comparison in recent times. Unprecedented measures are required to face this critical situation. Cancer patients are particularly exposed and may suffer from severe repercussions. The efforts of oncology professionals, including those working in the field of radiation oncology, should be targeted to increase the level of preparedness of the whole organization, adapt the decisional process to the current situation, decline the operational level following the principle of safety and good clinical sense, communicate and disseminate potentially useful information, and support staff, patients, and caregivers [11, 22, 23].
